# Glutamine metabolism and radiosensitivity: Beyond the Warburg effect

**DOI:** 10.3389/fonc.2022.1070514

**Published:** 2022-11-17

**Authors:** Ryan S. Alden, Mohammad Zahid Kamran, Bassel A. Bashjawish, Brittany A. Simone

**Affiliations:** Radiation Oncology Department, State University of New York (SUNY) Upstate Medical University, Syracuse, NY, United States

**Keywords:** cancer, glutamine (Gln), radiation, metabolism, radiosensitivity, telaglenastat, sirpiglenastat, immunotherapy

## Abstract

Mounting data suggest that cancer cell metabolism can be utilized therapeutically to halt cell proliferation, metastasis and disease progression. Radiation therapy is a critical component of cancer treatment in curative and palliative settings. The use of metabolism-based therapeutics has become increasingly popular in combination with radiotherapy to overcome radioresistance. Over the past year, a focus on glutamine metabolism in the setting of cancer therapy has emerged. In this mini-review, we discuss several important ways (DNA damage repair, oxidative stress, epigenetic modification and immune modulation) glutamine metabolism drives cancer growth and progression, and present data that inhibition of glutamine utilization can lead to radiosensitization in preclinical models. Future research is needed in the clinical realm to determine whether glutamine antagonism is a feasible synergistic therapy that can be combined with radiotherapy.

## 1 Introduction

Nearly 100 years ago, Otto Warburg demonstrated that cancer utilized more glucose and released more lactate than normal tissue under aerobic conditions (1). The understanding of altered metabolism as a cancer hallmark has accelerated in the last two decades (2, 3). More than 50% of cancer patients will receive radiation therapy, and improving response to therapy and decreasing toxicity is vital (4). There is growing interest in leveraging cancer metabolism to expand the therapeutic window. Calorie and carbohydrate restriction increase sensitivity to radiation *in vitro* and *in vivo*, but adoption in clinical trials has been slow (5), perhaps because weight loss is associated with poor cancer outcomes (5–9). Liu et al. recently summarized preclinical and early clinical studies regarding radiosensitization through inhibition of lactate metabolism (10). Over the last year, glutamine metabolism has continued to emerge as another important driver of resistance to anti-cancer therapies including radiation. Glutamine metabolism and transport plays a role in DNA damage repair (DDR), oxidative stress, epigenetic modification and immunosuppression. It thereby promotes tumor survival, growth and dissemination in addition to radioresistance. This mini-review summarizes recent investigations of glutamine manipulation for radiosensitization and anti-cancer therapy.

### 1.1 Glutamine in DNA damage repair and oxidative stress

**Figure 1:**
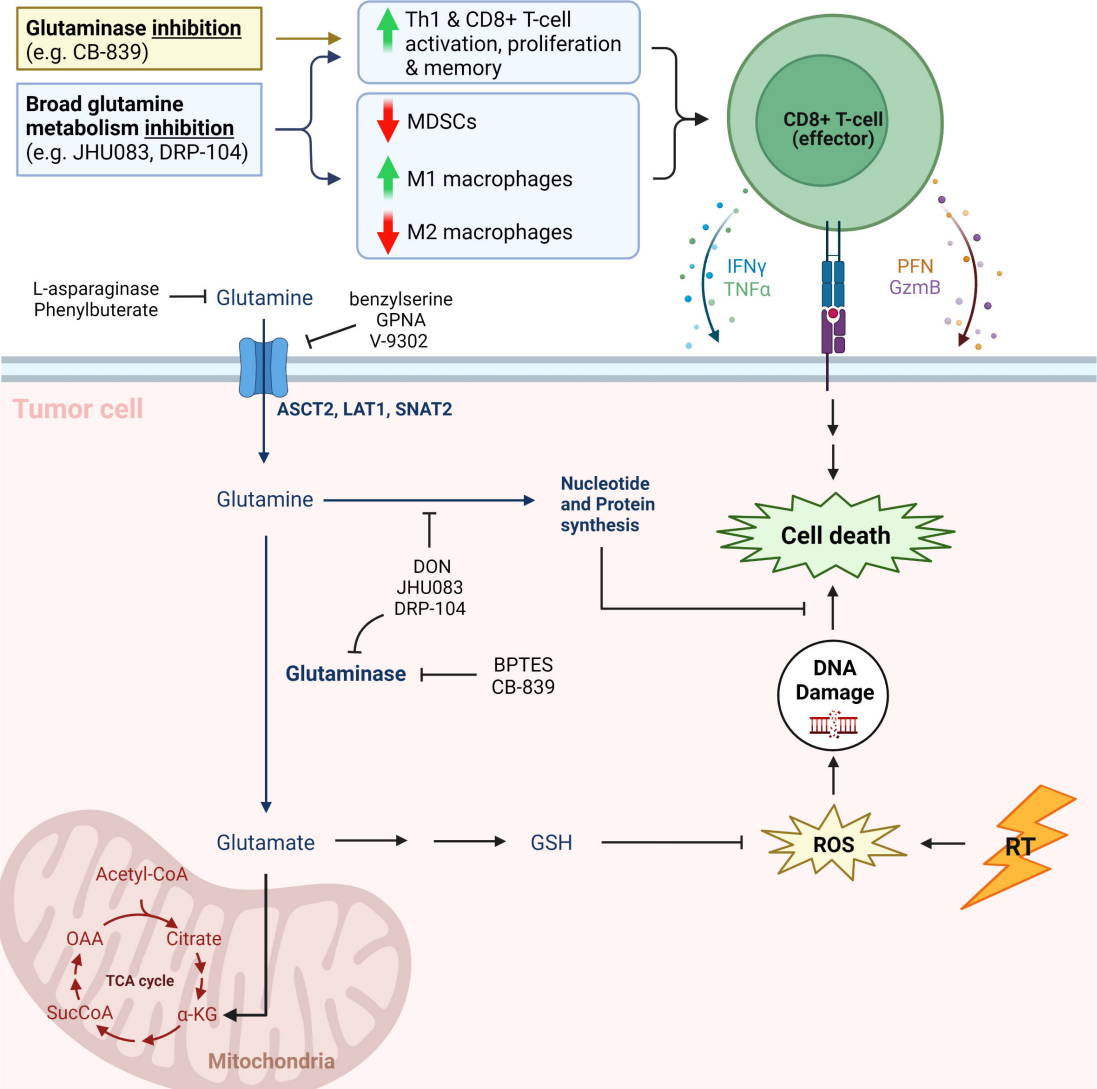
Simplified cartoon showing key aspects of glutamine metabolism and associated therapeutic strategies (11). CB-839, telaglenastat; DRP-104, sirpiglenastat; MDSCs, myeloid derived suppressor cells; IFNγ, interferon gamma; TNFΑ, tumor necrosis factor alpha; PFN, perforin; GzmB, Granzyme B; GPNA, L-γ-glutamyl-p-nitroanilide; DON, 6-diazo-5-oxo-L-norleucine; BPTES; bis-2-(5-phenylacetamido-1,3,4-thiadiazol-2-yl)ethyl sulfide; GSH, glutathione (reduced); ROS, reactive oxygen species; RT, radiation therapy; TCA cycle, tricarboxylic acid cycle; Α-KG, alpha ketoglutarate; SucCoA, Succinyl-CoA; OAA, oxaloacetate. Adapted from “Warburg Effect”, by BioRender.com.

Glutamine is critical to tumor metabolism and therefore is an attractive target for potential therapeutics (**Figure 1**). It contributes to DDR after ionizing radiation (IR) as a vital part of nucleotide synthesis (12). In addition, conversion of glutamine to glutathione *via* leads to increased capacity for DDR through free radical scavenging (12). As such, there are multiple small molecule inhibitors being investigated as potential radiosensitizers. These include V-9302, which antagonizes the c-Myc-regulated amino acid transporter ASCT2, as well as glutaminase (GLS) inhibitor CB-839. JHU083, a prodrug of the broad glutamine antagonist 6-diazo-5-oxo-L-norleucine (DON), disrupts NADP(H) and redox homeostasis in cancer cells, while decreasing hypoxia, a well-known contributor to radioresistance (13–15). Lastly, depletion of glutamine as a conditionally essential amino acid with the use of L-asparaginase (L-ASP) has been attempted as a means to induce cell cycle arrest and increase radiosensitivity (16, 17).

To date, several preclinical studies have been published utilizing CB-839 (Telaglenastat) to sensitize different cancers to IR (18, 19). Rashmi et al. have demonstrated that the use of CB-839 in PI3K- activated cervical cancers can independently affect cell survival in PTEN^-/-^ cervical cancer cell lines (20). Additionally, it was demonstrated that the combination of CB-839 with radiation provides improved tumor control in SiHa xenograft tumors, suggesting that CB-839 works synergistically with radiotherapy in cervical cancer. Similarly, after identifying that increased tumor GLS mRNA levels were associated with decreased survival in The Cancer Genome Atlas’ transcriptome database (p<0.03), Wicker et al. utilized CB-839 in combination with sublethal IR in a xenograft model of high GLS expressing, p16^-/-^, head and neck squamous cell carcinoma (21). Compared with control (vehicle treated) tumors, the size of radiation-treated, CB-839-treated and combination-treated tumors were 74.3%, 94.9% and 61.7%, respectively. Mechanistically, they showed that DNA damage assessed by γ-H2AX was significantly higher in combination-treated cells, and that this additive effect was reversible with N-acetyl-cysteine (21).

This concept is moving to the clinic in both cervical cancer and gliomas (**Table 1**). A phase I study investigating safety and tolerability of CB-839 with radiation and temozolomide in isocitrate dehydrogenase (IDH)- mutated grade II/III astrocytoma (NCT03528642) is currently active. The study will evaluate maximum tolerated dose and recommended phase II dose as primary outcomes as well as overall response rate (ORR) and clinical benefit rate as defined by Response Assessment in Neuro-Oncology (RANO) criteria. The same is being attempted in cervical cancer with NCT05521997, a randomized phase II trial of CB-839 in combination with cisplatin and radiation compared with standard chemoradiation. The primary endpoint in this study is progression-free survival at 24 months after therapy. Yang et al. and Lemberg et al. provide tables showing additional clinical trials involving CB-839 (17, 30).

**Table 1:** Selected preclinical studies and clinical trials exploring glutamine metabolism targets for cancer therapy.

Preclinical studies				
Target(s)	Inhibitor(s)	Model(s)	Selected Results	Reference(s)
GLS, gamma- glutamylcysteine synthetase, thioredoxin reductase	CB-839, BSO, AUR	CaSki, C33A, SiHa, SiHa PTEN^-/-^ cervical cancer cell lines CaSki and SiHa xenograft tumors	CB-839 induced oxidative stress, reduced cell proliferation, viability and surviving fraction in CaSki and SiHa PTEN^-/-^ cells; SiHa cells relatively resistant CB-839 & RT decreased cell survival *in vitro*, and decreased CaSki & SiHa tumor growth *in vivo*	Rashmi *Mol Cancer Ther* 2020 (20)
GLS	CB-839	CAL-27, HN5, FaDu HNSCC cell lines CAL-27 & HN5 xenografts	CB-839 & RT increased oxidative stress (8-oxoguanine) and DNA damage (γ-H2AX) in CAL-27 cells CB-839 & RT reduced tumor volume in xenografts	Wicker *Cancer Lett* 2021 (21)
glutamine	L-ASP	CWR22RV1, PC3, ARCaPM & ARCaPM-IR Pca cell lines:	glutamine depletion with L-ASP led to S phase accumulation due to G2/M block, and sensitized radioresistant cell line ARCaPM-IR to treatment	Thiruvalluvan *Cancers (Basel)* 2022 (22)
GLS, PD-1, PD-L1	CB-839, anti-PD-1, anti-PD-L1	Various Breast, NSCLC, lymphoma, myeloma, mesothelioma & ALL cell lines; CT26 colon cancer mouse model	CB-839 increases glutamine concentration and decreases glutamine metabolites in NSCLC cell lines, with minimal impact on T-cell activation or division CB-839 shows synergistic inhibition of tumor growth with anti-PD-1 or anti-PD-L1 inhibition *in vivo*	Gross *Cancer Res* 2016 (23)
GLS	CB-839, anti-CTLA4, anti-PD-1	Melanoma: patient derived cell lines & TILs, A375HG cells, B16-F10 xenografts Yummer 1.7 mouse model	CB-839 in tumor cells cocultured with TILs increased cleaved caspase-3 CB-839 enhanced T-cell proliferation and activation in an *in vivo* adoptive T-cell therapy model CB-839 enhances anti-tumor effect of IO	Varghese *Mol Cancer Ther* 2020 (24)
glutamine metabolism	JHU083, anti-PD-1	mice models: MC38 & CT26 colon cancer, EL-4 lymphoma, B16 melanoma	decreased tumor growth & improved survival with JHU083 treatment in all models, with some complete responses & rejection of tumor re-challenge JHU083 pushes TILs to a long-lived, memory-like, proliferative and activated phenotype	Leone *Science* 2019 (13)
glutamine metabolism	JHU083 (prodrug of DON), anti-CTLA4, anti-PD-1	4T1 triple-negative breast cancer model EO771 triple-negative breast cancer model	JHU083 inhibits 4T1 tumor growth and metastasis; enhanced response to checkpoint blockade JHU083 reduced MDSCs, decreased secretion of MDSC recruitment & growth factors, reprogrammed TAMs to proinflammatory phenotype	Oh *J Clin Invest* 2020 (25)
glutamine metabolism	DRP-104 (prodrug of DON), anti-PD-1, anti-PD-L1, anti-CTLA4	CT26 & MC38 colon cancer models H22 hepatocellular cancer model	DRP-104 increased T, NK, NKT cells and M1-TAMs and decreased MDSCs; also inhibited tumor growth, led to durable responses and rejection of rechallenge	Yokoyama *Mol Cancer Ther* 2022 (26) Yokoyama *J Immunother Cancer* 2019 (27) Yokoyama *Cancer Res* 2020 (28)
Clinical studies				
Target(s)	Treatment	Diagnosis	Status	Trial Number
GLS	CB-839 with RT and temozolomide	IDH-mutant astrocytoma grade II/III	Phase 1b trial: Active, not recruiting	NCT03528642
GLS	RT and cisplatin *vs.* CB-839 with RT and cisplatin	FIGO III-IV cervical cancer	Randomized Phase II trial: Not yet recruiting	NCT05521997
GLS, PD-1	CB-839 with nivolumab	Advanced melanoma, NSCLC with prior IO; Advanced RCC with or without prior IO	Phase I/II trial: recruitment completed 04/2020	NCT02771626, Meric-Bernstam *SITC* 2017 (29)

GLS, glutaminase; CB-839, telaglenastat; BSO, L-buthionine sulfoximine; AUR, Auranofin; RT, radiation therapy; HNSCC, head and neck squamous cell carcinoma; L-ASP, L-asparaginase; DON, 6-diazo-5-oxo-L-norleucine; NSCLC, non-small cell lung cancer; ALL, acute lymphoblastic leukemia; IO, immunotherapy; TIL, tumor infiltrating lymphocyte; MDSCs, myeloid derived suppressor cells; TAMs, tumor associated macrophages; DRP-104, sirpiglenastat; FIGO, Federation of Gynecology and Obstetric; RCC, renal cell carcinoma.

Depleting extracellular glutamine with L-ASP provides another avenue of potential therapeutic benefit. Targeting extracellular glutamine circumvents the non-specificity and toxicity of glutamine uptake inhibitors (24, 31). Additionally, inhibition of one specific glutamine uptake receptor can lead to upregulation of alternate receptors, rendering these therapies ineffective. Thiruvalluvan et al. demonstrated that the use of L-ASP in prostate cancer leads to cell cycle arrest, thereby increasing sensitivity to DNA damage (22). Treatment of 22Rv1 cells with L-ASP lead to downregulation of CDK1, CCNB1 and PCNA with elevation of p21 in cells treated with radiation. In this study, a radioresistant prostate cancer cell line was created (ARCAP_M_-IR). Exposure to L-ASP sensitized these cells to radiation treatment compared with the parent line (ARCAP_M_) (22).

### 1.2 Alpha-ketoglutarate and the epigenetic landscape of tumors

Once transported intracellularly, glutamine can be trafficked to the mitochondria of cancer cells and metabolized to glutamate, which impacts production of alpha-ketoglutarate (ΑKG) through the tricarboxylic acid (TCA) cycle. This ΑKG is then transported out of the mitochondria and modifies expression of key proteins linked to cancer growth and progression. Histone and DNA methylation is directly affected by ΑKG (32, 33). It is by this process ΑKG is posited to affect stemness in cancer cells, and its concentration within cells can drive progression and metastasis. Tran et al. recently demonstrated that ΑKG promotes hypomethylation of DNA histone H3K4me3, which targets several Wnt pathway genes in colorectal cancer (33). Likewise, ΑKG affects radiosensitivity in the setting of IDH mutations. In IDH- mutated tumors, there is an abundance of ΑKG leading to accumulation of a by-product of the Krebs cycle, 2-hydroxyglutarate (2HG), which ultimately affects methylation of enzymes responsible for DNA double strand break (DSB) repair (34). This aberration in DSB repair leads to increased radiosensitivity as seen in IDH mutated gliomas (34, 35).

Histone and DNA methylation are directly affected by ΑKG levels through ΑKG-dependent enzymes including jumonji domain-containing histone demethylases and Tet proteins that modify DNA methylation. Mukha et al. demonstrate that the radiosensitivity of glutamine-dependent tumors such as prostate cancer can be manipulated through this pathway (36). Homologous recombination repair (HRR) aberrations have been linked to impaired histone demethylation by ΑKG-dependent dioxygenases KDM4A and KDM4B. It was hypothesized by Sulkowski et al. that H3K9 methylation is directly affected by 2HG, and that high concentrations of this metabolite cause impairment of DNA DSB localization (37). This ultimately implies that accumulation of 2HG in the context of radiation could cause sensitization through hypermethylation of H3K9 (37). This defect in HRR caused by 2HG accumulation mimics BRCA1/2-deficient tumors as it has been shown to sensitize cells to PARP inhibitors (37). In line with this concept, the function of KDMs as modulated by oncometabolites has been linked to radioresistance in lung cancer (38).

Taken together, inherent mutations of metabolic pathways in cancer cells such as IDH mutations as well as accumulation of oncometabolites such as ΑKG within tumors create a diverse metabolic tumor microenvironment (TME) that directly impacts DSB repair. This creates an opportunity to use these vulnerabilities to therapeutic advantage in combination with radiotherapy.

### 1.3 Glutamine and anticancer immunity

The interaction between radiotherapy and the immune system is complex and continues to be elucidated (39–44). Radiation promotes tumor immunogenicity through increased expression of chemokines such as CXCL9, CXCL10 and CXCL16 which lead to T-cell recruitment (40, 42, 43). Simultaneously, radiation leads to release of tumor antigens, expression of death receptors, MHC class I proteins, and costimulatory molecules that facilitate T-cell- mediated killing of tumor cells (40, 44–46). Radiation also enhances PD-L1 expression, which holds T-cell reactivity in check (40, 47). Radiation can induce differentiation of Tregs and myeloid-derived suppressor cells (MDSCs), which hinder anti-tumor immunity (39, 40, 48). Radiation therapy also has mixed effects on the innate immune response, as it contributes to M1, pro-inflammatory anti-tumor macrophages, but also to M2, immunosuppressive and pro-tumor macrophages (39, 40). Therefore, the ideal metabolic strategy for radiosensitization would also contribute to the anti-tumor immune response and ameliorate the immunosuppressive effects of radiation. Recent evidence suggests that inhibition of glutamine metabolism meets these objectives. Multiple authors have shown that inhibition of glutamine metabolism impacts glutamine-dependent tumor cells and immune cells differently, ultimately bolstering innate and adaptive anti-tumor response (13, 23–25, 29, 30).

#### 1.3.1 Glutamine and T-cell activation

Glutamine metabolism plays an important role in T-cell activation and proliferation, as a precursor for biosynthesis and source of ΑKG for the TCA cycle and ATP production (17, 30, 49). Glutamine induces a dose-dependent increase in proliferation of stimulated T-cells *in vitro* (23, 24). On the surface therefore, it may seem that glutamine inhibition would frustrate anticancer immunity, but recent investigation shows otherwise.

Johnson et al. demonstrated that in effector T-cell populations with transient GLS deficiency, IL2 promotes Th1 phenotype and CD8 T-cell function, suggesting that inhibition of glutamine metabolism enhances an anti-tumor immune composition (24, 50). Aberrant tumor metabolism, which also upregulates glutamine metabolism for biosynthesis and anaplerosis, depletes glutamine in the tumor environment (13, 24, 30). Inhibition of GLS with CB-839, or of glutamine metabolism broadly with JHU083 or DRP-104 (Sirpiglenastat, another pro-drug of DON), restores balance between tumor and T-cell glutamine utilization. Multiple authors have shown that CB-839, JHU083 and DRP-104 reduce tumor glutamine consumption and increase glutamine concentrations systemically and in the TME, providing T-cells with greater access to this conditionally essential amino acid (13, 17, 23, 24, 26, 29). CB-839 also has minimal impact on T-cell proliferation and acceptable toxicity profile, unlike the non-specific inhibitor DON (23, 24). Leone et al. found that JHU083 increased proliferation and activation markers in CD8+ T-cells in mice harboring MC38 colon cancer, and selective activation of this prodrug in the TME mitigates toxicity observed with DON (13).

Gross et al. demonstrated in a syngeneic CT26 colon cancer mouse model that combination CB-839 and anti-PD-1 or anti-PD-L1 therapy enhanced tumor regression and improved survival compared to control or monotherapy (23). These benefits were reversed with depletion of CD8+ T-cells from the tumors, supporting the hypothesis that GLS inhibition with CB-839 enhances CD8+ T-cell activity in the TME when administered with checkpoint inhibitors (23). These results led to the phase I/II trial CX-839-004; CB-839 with nivolumab in advanced melanoma, renal cell carcinoma (RCC), or non-small cell lung cancer. The trial completed recruitment in 2020. Preliminary results showed no toxicity above nivolumab alone, and three patients with melanoma and progression on prior immunotherapy achieved objective response (29).

Building on the potential to revitalize checkpoint inhibitor response in the pre-treated setting, Varghese et al. report promising preclinical results in treatment-naïve melanoma models (24). In mice vaccinated with melanoma-specific CD8+ T-cells and stimulatory molecules, CB-839 increased T-cell proliferation and activation compared to vehicle control (24). T-cell vaccination with CB-839 treatment led to the greatest decrease in B16 xenograft tumor growth *vs.* monotherapy or control. Mouse survival was 100% at 35 days in the combination group (24). These results suggest clinical study of GLS inhibition together with T-cell vaccines or other T-cell therapies is warranted.

Varghese et al. also tested CB-839 together with immune-checkpoint inhibitors in a BRAF V600E, high mutational burden melanoma model. Anti-PD-1 or anti-CTLA4 therapy inhibited tumor growth, in keeping with prior findings (24, 51). CB-839 monotherapy had no effect on tumor growth, but combination with anti-PD-1 or anti-CTLA4 showed synergistic effects, with triple therapy leading to apparent complete response at 29 days (24).

JHU083 has also shown exciting results, with improved tumor control and survival in mouse models of several tumor types (13). JHU083 with concurrent anti-PD-1 therapy in a MC38 mice showed complete response rate of ≥90%, compared to no complete responses with anti-PD-1 monotherapy (13). Lack of single-agent activity is a criticism some have leveled against CB-839 (13, 23, 52). In contrast, in one experiment, 2 of 5 mice with MC38 tumors had complete response to JHU083 monotherapy maintained >80 days after tumor injection (13). Additionally, 13 of 15 animals with complete response to JHU083 monotherapy rejected tumor upon rechallenge. These results indicate JHU083 may have single agent utility. As with the CB-839 experience above, depletion of T-cells demonstrated that JHU083 efficacy relied on CD8+ T-cell activity (13). Based on these data, along with RNA-seq and gene set enrichment analysis (GSEA) of tumor infiltrating lymphocytes (TILs), Leone et al. concluded that JHU083 pushes TILs toward a long-lived, memory-like, proliferative and highly activated effector phenotype (13, 17).

DRP-104 produced similar results in mice harboring colon and hepatocellular model tumors (26–28, 30). Yokoyama et al. found that DRP-104 tumor growth inhibition at day 12 in CT26-bearing mice was 48% with anti-PD-1, 90% with DRP-104 and 94% with combination (26, 27). In MC38-bearing mice, DRP-104 monotherapy led to growth inhibition of 96-101% and increase in median survival from 13 days with vehicle control to 31-38 days with DRP-104 (26). Eight of 16 CT26 mice treated with combination DRP-104 and anti-PD-L1 were tumor free at day 77, and all eight rejected tumor rechallenge (27). In mice with H22 hepatocellular tumors, DRP-104 at low (45 days) or high dose (47 days) alone and in combination with anti-PD-L1 blockade (76.5 or 94 days) significantly extended survival compared to vehicle (27.5 days) and anti-PD-L1 (29.5 days) alone (26, 27). There were 50% durable cures in high dose combination-treated mice (26, 27).

These data further support GLS and glutamine inhibition in combination with checkpoint blockade in the clinical, treatment-naïve setting. Additional study in combination with radiation also seems promising, since restoration of T-cell response could turn radioresistance into radiosensitivity.

#### 1.3.2 Glutamine and M2 macrophages

While cancer cell metabolism hoards nutrients such as glutamine, the TME is further stripped of resources by the activity of M2 macrophages and MDSCs. Tumor-associated macrophages (TAMs) contribute to cancer progression and metastasis by promoting angiogenesis, invasion, motility, intravasation and extravasation, while also curating an immunosuppressive environment (25, 39, 53, 54). M2 TAMs and MDSCs express immune checkpoint molecules like PD-L1 to attenuate T-cell response, and metabolic enzymes which deplete nutrients and smother T-cell proliferation. Oh et al. report \ inhibition of glutamine metabolism with JHU083 promoted generation of antitumor, proinflammatory TAMs and reduced generation and recruitment of MDSCs (25). Similarly, Yokoyama et al. report that DRP-104 treatment increased M1-polarized TAMs and decreased MDSCs (26, 28).

4T1 is a triple-negative breast cancer model that is resistant to immune checkpoint blockade due to abundant suppressive TAMs and MDSCs (25, 55). JHU083 showed significant inhibition of 4T1 tumor growth in mice compared with vehicle, anti-PD1, anti-CTLA4 or combination checkpoint blockade (25). JHU083 also significantly inhibited lung metastasis. JHU083 enhanced checkpoint blockade activity in EO771 immunotherapy-sensitive tumors, and sensitized 4T1 immunotherapy-resistant tumors to checkpoint blockade (25).

Oh et al. demonstrated that modulation of TAMs and MDSCs is responsible for these effects. JHU083 decreased MDSCs at the primary tumor site and in the lungs (common metastatic site). This was accompanied by decreased MDSC recruitment and growth factors such as M-CSF, GM-CSF and G-CSF. Finally, Oh et al. found through RNA-seq and GSEA that JHU083 reprogrammed TAMs promoting an M1, antitumor phenotype *via* down regulation of immunosuppressive genes such as *Il10* and *Nos2*, with upregulation of lysosome, Toll-like receptor and proinflammatory gene transcription.

These data suggest broad glutamine antagonism with JHU083 or DRP-104 may increase radiosensitivity by favoring an antitumor immune response, but clinical study including combination with radiation is necessary.

#### 1.3.3 Sequencing of combination therapy

Sequencing of immunotherapy and radiotherapy is an area of ongoing research; full exploration of this topic is beyond the scope of this mini-review (56, 57). Optimal timing depends on the mechanism of immunotherapy agent used and the fraction size of radiation (57). Notably, secondary analysis of KEYNOTE-001 found a survival benefit with pembrolizumab in patients previously treated with radiotherapy *vs* no prior radiotherapy (56, 58). As such, several clinical studies have specified immunotherapy within 3-84 days after radiation (59–61). Radiation can begin before, during or after immunotherapy in the ongoing A082002 trial in metastatic NSCLC, as long as it begins within 60 days of registration (NCT04929041). We hypothesize that radiation followed by or concurrent with immunotherapy, rather than starting with immunotherapy, will lead to optimal results by exposing tumor antigens and utilizing radiation-induced increase in PD-L1 expression to fuel immunotherapy response (56, 62). This hypothesis seems to be supported by the survival benefit from pembrolizumab after radiation in the PEMBRO-RT trial, but only in patients with negative PD-L1 at baseline, where perhaps PD-L1 expression was induced by radiation (59).

Adding glutamine antagonists leaves additional uncertainty regarding the best sequence, but Leone et al. report that “concurrent, not sequential” administration of JHU083 and anti-PD-1 was most effective (13). Most pre-clinical and clinical studies have initiated glutamine antagonists concomitantly with immunotherapy (23–25, 29), others have used concomitant and sequential approaches without direct comparison of the two (24, 27, 28). Wicker et al. introduced CB-839 two days before radiating HNSCC cells (21).

### 1.4 Personalized medicine: Identifying glutamine-dependent tumors that may benefit from inhibition of glutamine metabolism

Multiple cancer subtypes appear to be sensitive to glutamine inhibition. Gross et al. show cell death at glutamine deficit and dose-dependent growth enhancement with increasing glutamine concentrations in breast cancer, NSCLC, lymphoma, myeloma, mesothelioma and ALL cell lines (23). Sensitivity to glutamine metabolism in cervical cancer, melanoma, RCC, IDH-mutant glioma, HNSCC, colon cancer and hepatocellular cancer has been demonstrated by other authors discussed in this review (**Table 1**) (13, 17–30, 34, 50). One might hypothesize that PTEN-mutated endometrial cancers and IDH-mutant AML are sensitive given their genotype (63, 64). However, not all tumors are addicted to glutamine metabolism, and Yuneva et al. point out “the metabolic profile of tumors depends on both the responsible genetic lesion and tissue type” (65). MYC-driven liver tumors are dependent on glutamine catabolism, while MET-driven liver tumors are not dependent on glutamine catabolism (65). Davidson et al. found that *KRAS* G12D NSCLCs were not dependent on glutamine metabolism (52). Therefore, an understanding of what tumors may be sensitive to glutamine inhibition must look beyond simply the tissue of origin. Patient- derived xenograft experiments that incorporate sequencing or immunohistochemistry may be required to understand which tumors are ultimately sensitive and provide personalized cancer care.

## 2 Discussion

Glutamine metabolism contributes to cancer growth, progression and resistance to therapy. As a key player in DNA repair, epigenetic modification and reduction in oxidative stress, glutamine metabolism in cancer cells also increases radioresistance. Furthermore, glutamine use in cancer cells, TAMs and MDSCs contributes to an immunosuppressive TME which decreases the efficacy of immunotherapy and radiotherapy. Inhibition of glutamine metabolism increases oxidative stress in cancer cells, sensitizes radioresistant tumors to radiation *in vitro* and *in vivo* (18–20) and enhances innate and adaptive anti-tumor immunity (13, 17, 23, 25, 27–29, 50). Recent reports disclose several avenues for leveraging inhibition of, or abnormalities in, glutamine metabolism to widen the therapeutic window for anti-cancer therapy including radiotherapy (13, 17–21, 23–30, 34, 50). This approach seems most promising in tumors with: high GLS expression such as some p16-negative HNSCC (21), DNA repair deficit such as IDH-mutant glioma (19, 34), or PTEN mutation/PI3K activation (20). Specifically, we anticipate combination with CB-839 will enhance the efficacy of standard of care chemoradiation in IDH-mutant GII/III astrocytoma (19, 34). CB-839 will likely increase DNA damage from increased oxidative stress and cause further impairment of DNA repair leading to enhanced tumor killing (19, 35). We also hypothesize CB-839 in combination with chemoradiation will improve outcomes in cervical cancer, particarly in PTEN mutant/PI3K activated cancers (20). By extension, the combination of CB-839 and radiation may also improve outcomes if studied in endometrial cancer, which can also harbor PTEN mutations (63). There may also be hope for radiosensitizing classically radioresistant cancers such as RCC through concurrent radiation and CB839, JHU083 or DRP-104 (29). Enhancing the radiosensitivity of these tumors may allow for deescalation of radiotherapy dose and reduced toxicity. Further combination with immunotherapy is also an attractive avenue, with optimal sequence of therapies an ongoing area of research (56, 57).

Ultimately, additional clinical research is necessary to determine feasibility and efficacy in combination with radiotherapy, and to determine which drug for targeting the glutamine pathway yields optimal clinical results (17).

## Author contributions

RA, MK, BB, and BS, drafting the article, critical revision of the article, and final approval of the version to be published. BS, conception or design of the work. All authors contributed to the article and approved the submitted version.

## Conflict of interest

The authors declare that the research was conducted in the absence of any commercial or financial relationships that could be construed as a potential conflict of interest.

## Publisher’s note

All claims expressed in this article are solely those of the authors and do not necessarily represent those of their affiliated organizations, or those of the publisher, the editors and the reviewers. Any product that may be evaluated in this article, or claim that may be made by its manufacturer, is not guaranteed or endorsed by the publisher.

## References

[1] Warburg O Posener K Negelein E . Über den stoffwechsel der carcinomzelle. Biochem Zeitschr (1924) 152:309–44.

[2] Hanahan D Weinberg RA . The hallmarks of cancer. Cell (2000) 100(1):57–70. doi: 10.1016/S0092-8674(00)81683-9 10647931

[3] Hanahan D Weinberg RA . Hallmarks of cancer: The next generation. Cell (2011) 144(5):646–74. doi: 10.1016/j.cell.2011.02.013 21376230

[4] Delaney G Jacob S Featherstone C Barton M . The role of radiotherapy in cancer treatment: Estimating optimal utilization from a review of evidence-based clinical guidelines. Cancer (2006) 104(6):1129–37. doi: 10.1002/cncr.21324 16080176

[5] Icard P Ollivier L Forgez P Otz J Alifano M Fournel L . Perspective: Do fasting, caloric restriction, and diets increase sensitivity to radiotherapy? a literature review. Adv Nutr (2020) 11(5):1089–101. doi: 10.1093/advances/nmaa062 PMC7490158 32492154

[6] Tsang NM Pai PC Chuang CC Chuang WC Tseng CK Chang KP . Overweight and obesity predict better overall survival rates in cancer patients with distant metastases. Cancer Med (2016) 5(4):665–75. doi: 10.1002/cam4.634 PMC4831285 26811258

[7] Gannavarapu BS Lau SKM Carter K Cannon NA Gao A Ahn C . Prevalence and survival impact of pretreatment cancer-associated weight loss: A tool for guiding early palliative care. J Oncol Pract (2018) 14(4):e238–e50. doi: 10.1200/JOP.2017.025221 PMC5951294 29466074

[8] Languis JAE van Dijk AM Doornaert P Kruizenga HM Langendijk JA Leemans CR . More than 10% weight loss in head and neck cancer patients during radiotherapy is independently associated with deterioration in quality of life. Nutr Cancer (2012) 65(1):76–83. doi: 10.1080/01635581.2013.741749 23368916

[9] Languis JAE Bakker S Rietveld DHF Kruizenga HM Langendijk JA Weijs PJM . Critical weight loss is a major prognostic indicator for disease-specific survival in patients with head and neck cancer receiving radiotherapy. Br J Cancer (2013) 109(5):1093–9. doi: 10.1038/bjc.2013.458 PMC3778304 23928661

[10] Liu KX Everdell E Pal S Haas-Kogan DA Milligan MG . Harnessing lactate metabolism for radiosensitization. Front Oncol (2021) 11. doi: 10.3389/fonc.2021.672339 PMC8343095 34367959

[11] Warburg effect. BioRender.com (2022). Available at: https://app.biorender.com/biorender-templates.

[12] Pathways of human metabolism, version 10.16. Stanford School of Medicine, Department of Biochemistry (2016). Available at: https://metabolicpathways.stanford.edu/, https://help.biorender.com/en/articles/5011393-using-a-biorender-template.

[13] Leone RD Zhao L Englert JM Sun I-H Oh M-H Sun I-H . Glutamine blockade induces divergent metabolic programs to overcome tumor immune evasion. Science (2019) 366(6468):1013–21. doi: 10.1126/science.aav2588 PMC7023461 31699883

[14] Bush RS Jenkin RD Allt WE Beale FA Bean H Dembo AJ . Definitive evidence for hypoxic cells influencing cure in cancer therapy. Br J Cancer Suppl (1978) 3:302–6. PMC2149436 277250

[15] Brown JM . Sr 4233 (Tirapazamine): A new anticancer drug exploiting hypoxia in solid tumours. Br J Cancer (1993) 67(6):1163–70. doi: 10.1038/bjc.1993.220 PMC1968495 8512801

[16] Avramis VI . Asparaginases: Biochemical pharmacology and modes of drug resistance. Anticancer Res (2012) 32(7):2423–37. 22753699

[17] Yang W-H Qiu Y Stamatotos O Janowitz T Lukey MJ . Enhancing the efficacy of glutamine metabolism inhibitors in cancer therapy. Trends Cancer (2021) 7(8):790–804. doi: 10.1016/j.trecan.2021.04.003 34020912 PMC9064286

[18] Boysen G Jamshidi-Parsian A Davis MA Siegel ER Simecka CM Kore RA . Glutaminase inhibitor cb-839 increases radiation sensitivity of lung tumor cells and human lung tumor xenografts in mice. Int J Radiat Biol (2019) 95(4):436–42. doi: 10.1080/09553002.2018.1558299 PMC6622448 30557074

[19] McBrayer SK Mayers JR DiNatale GJ Shi DD Khanal J Chakraborty AA . Transaminase inhibition by 2-hydroxyglutarate impairs glutamate biosynthesis and redox homeostasis in glioma. Cell (2018) 175(1):101–16.e25. doi: 10.1016/j.cell.2018.08.038 30220459 PMC6219629

[20] Rashmi R Jayachandran K Zhang J Menon V Muhammad N Zahner M . Glutaminase inhibitors induce thiol-mediated oxidative stress and radiosensitization in treatment-resistant cervical cancers. Mol Cancer Ther (2020) 19(12):2465–75. doi: 10.1158/1535-7163.MCT-20-0271 PMC8208465 33087507

[21] Wicker CA Hunt BG Krishnan S Aziz K Parajuli S Palackdharry S . Glutaminase inhibition with telaglenastat (Cb-839) improves treatment response in combination with ionizing radiation in head and neck squamous cell carcinoma models. Cancer Lett (2021) 502:180–8. doi: 10.1016/j.canlet.2020.12.038 PMC7897292 33450358

[22] Thiruvalluvan M Billet S Bhowmick NA . Antagonizing glutamine bioavailability promotes radiation sensitivity in prostate cancer. Cancers (Basel) (2022) 14(10):2491. doi: 10.3390/cancers14102491 35626095 PMC9139225

[23] Gross M Chen J Englert J Janes J Leone R MacKinnon A . Abstract 2329: Glutaminase inhibition with cb-839 enhances anti-tumor activity of pd-1 and pd-L1 antibodies by overcoming a metabolic checkpoint blocking T cell activation. Cancer Res (2016) 76(14_Supplement):2329. doi: 10.1158/1538-7445.AM2016-2329

[24] Varghese S Pramanik S Williams LJ Hodges HR Hudgens CW Fischer GM . The glutaminase inhibitor cb-839 (Telaglenastat) enhances the antimelanoma activity of T-Cell-Mediated immunotherapies. Mol Cancer Ther (2021) 20(3):500–11. doi: 10.1158/1535-7163.MCT-20-0430 PMC7933078 33361272

[25] Oh M-H Sun I-H Zhao L Leone RD Sun I-M Xu W . Targeting glutamine metabolism enhances tumor-specific immunity by modulating suppressive myeloid cells. J Clin Invest (2020) 130(7):3865–84. doi: 10.1172/JCI131859 PMC7324212 32324593

[26] Yokoyama Y Estok TM Wild R . Sirpiglenastat (Drp-104) induces antitumor efficacy through direct, broad antagonism of glutamine metabolism and stimulation of the innate and adaptive immune systems. Mol Cancer Ther (2022) 21(10):1561–72. doi: 10.1158/1535-7163.MCT-22-0282 35930753

[27] Yokoyama Y Nedelcovych M Wild R . P497 drp-104, a novel broad acting glutamine antagonist, induces distinctive immune modulation mechanisms and synergistic efficacy in combination with immune checkpoint blockade. 34th annual meeting & pre-conference programs of the society for immunotherapy of cancet (Sitc 2019): Part 1. J Immunother Cancer (2019) 7(suppl 1):282.31694725

[28] Yokoyama Y Wild R . (2020). Broad acting glutamine antagonism remodels the tumor microenvironment, induces distinctive immune modulation, and synergizes with immune checkpoint blockade [Abstract], in: Proceedings of the Annual Meeting of the American Association for Cancer Research 2020, Philadelphia, PA: AACR, 2020 Apr 27-28 and Jun 22-24. p. 5607.

[29] Meric-Bernstam F Gordon M Tykodi S Lam E Vaishampayan U Chaves J . (2017). Cx-839-004: A phase 1/2 study of cb-839, a first-in-Class glutaminase inhibitor, combined with nivolumab in patients with advanced melanoma (Mel), renal cell carcinoma (Rcc), or non-small cell lung cancer (Nsclc), in: Society for Immunotherapy of Cancer Annual Meeting, National Harbor, MD, USA, November 8-12, 2017.

[30] Lemberg KM Gori SS Tsukamoto T Rais R Slusher BS . Clinical development of metabolic inhibitors for oncology. J Clin Invest (2022) 132(1):e148550. doi: 10.1172/JCI148550 34981784 PMC8718137

[31] Catane R Hoff DDV Glaubiger DL Muggia FM . Azaserine, don, and azotomycin: Three diazo analogs of l-glutamine with clinical antitumor activity. Cancer Treat Rep (1979) 63(6):1033–8. 380801

[32] Carey BW Finley LWS Cross JR Allis CD Thompson CB . Intracellular Α-ketoglutarate maintains the pluripotency of embryonic stem cells. Nature (2015) 518(7539):413–6. doi: 10.1038/nature13981 PMC4336218 25487152

[33] Tran TQ Hanse EA Habowski AN Li H Ishak Gabra MB Yang Y . Α-ketoglutarate attenuates wnt signaling and drives differentiation in colorectal cancer. Nat Cancer (2020) 1(3):345–58. doi: 10.1038/s43018-020-0035-5 PMC7442208 32832918

[34] Chen L-L Xiong Y . Tumour metabolites hinder DNA repair. Nature (2020) 582(7813):492–4. doi: 10.1038/d41586-020-01569-1 PMC7576997 32572248

[35] Cairncross JG Wang M Jenkins RB Shaw EG Giannini C Brachman DG . Benefit from procarbazine, lomustine, and vincristine in oligodendroglial tumors is associated with mutation of idh. J Clin Oncol (2014) 32(8):783–60. doi: 10.1200/JCO.2013.49.3726 PMC3940537 24516018

[36] Mukha A Kahya U Linge A Chen O Löck S Lukiyanchuk V . Gls-driven glutamine catabolism contributes to prostate cancer radiosensitivity by regulating the redox state, stemness and Atg5-mediated autophagy. Theranostics (2021) 11(16):7844–68. doi: 10.7150/thno.58655 PMC8315064 34335968

[37] Sulkowski PL Oeck S Dow J Economos NG Mirfakhraie L Liu Y . Oncometabolites suppress DNA repair by disrupting local chromatin signalling. Nature (2020) 582(7813):586–91. doi: 10.1038/s41586-020-2363-0 PMC7319896 32494005

[38] Kuo K-T Huang W-C Bamodu OA Lee W-H Wang C-H Hsiao M . Histone demethylase Jarid1b/Kdm5b promotes aggressiveness of non-small cell lung cancer and serves as a good prognostic predictor. Clin Epigenet (2018) 10:107. doi: 10.1186/s13148-018-0533-9 PMC6085612 30092824

[39] Shi X Shiao SL . The role of macrophage phenotype in regulating the response to radiation therapy. Transl Res (2018) 191:64–80. doi: 10.1016/j.trsl.2017.11.002 29175267 PMC6018060

[40] Le Q-T Shirato H Giaccia AJ Koong AC . Emerging treatment paradigms in radiation oncology. Clin Cancer Res (2015) 21(15):3393–401. doi: 10.1158/1078-0432.CCR-14-1191 PMC4526434 25991820

[41] Byron B Weichselbaum RR . Radiation as an immune modulator. Semin Radiat Oncol (2013) 2013(4):273–80. doi: 10.1016/j.semradonc.2013.05.009 24012341

[42] Lugade AA Sorensen EW Gerber SA Moran JP Frelinger JG Lord EM . Radiation-induced ifn-gamma production within the tumor microenvironment influences antitumor immunity. J Immunol (2008) 180(5):3132–9. doi: 10.4049/jimmunol.180.5.3132 18292536

[43] Matsumura S Wang B Kawashima N Braunstein S Badura M Cameron TO . Radiation-induced Cxcl16 release by breast cancer cells attracts effector T cells. J Immunol (2008) 181(5):3099–107. doi: 10.4049/jimmunol.181.5.3099 PMC2587101 18713980

[44] Formenti SC Demaria S . Combining radiotherapy and cancer immunotherapy: A paradigm shift. J Natl Cancer Inst (2013) 105(4):256–65. doi: 10.1093/jnci/djs629 PMC3576324 23291374

[45] Reits E Hodge JW Herberts CA Groothuis TA Chakraborty M Wansley EK . Radiation modulates the peptide repertoire, enhances mhc class I expression, and induces successful antitumor immunotherapy. J Exp Med (2006) 203(5):1259–71. doi: 10.1084/jem.20052494 PMC3212727 16636135

[46] Ifeadi V Garnett-Benson C . Sub-Lethal irradiation of human colorectal tumor cells imparts enhanced and sustained susceptibility to multiple death receptor signaling pathways. PLoS One (2012) 7(2):e31762. doi: 10.1371/journal.pone.0031762 22389673 PMC3289623

[47] Deng L Liang H Byron B Beckett M Darga T Weichselbaum RR . Irradiation and anti–Pd-L1 treatment synergistically promote antitumor immunity in mice. J Clin Invest (2014) 124(2):687–95. doi: 10.1172/JCI67313 PMC3904601 24382348

[48] Kachikwu EL Iwamoto KS Liao Y-P DeMarco JJ Agazaryan N Economou JS . Radiation enhances regulatory T cell representation. Int J Radiat Oncol Biol Phys (2011) 81(4):1128–35. doi: 10.1016/j.ijrobp.2010.09.034 PMC3117954 21093169

[49] Wang R Green DR . Metabolic reprogramming and metabolic dependency in T cells. Immunol Rev (2012) 249(1):14–26. doi: 10.1111/j.1600-065X.2012.01155.x 22889212 PMC3422760

[50] Johnson MO Wolf MM Madden MZ Andrejeva G Sugiura A Contreras DC . Distinct regulation of Th17 and Th1 cell differentiation by glutaminase-dependent metabolism. Cell (2018) 175(7):1780–95. doi: 10.1016/j.cell.2018.10.001 PMC6361668 30392958

[51] Wang J Perry CJ Meeth K Thakral D Damsky W Micevic G . Uv-induced somatic mutations elicit a functional T cell response in the Yummer1.7 mouse melanoma model. Pigment Cell Melanoma Res (2017) 30(4):428–35. doi: 10.1111/pcmr.12591 PMC5820096 28379630

[52] Davidson SM Papagiannakopoulos T Olenchock BA Heyman JE Keibler MA Luengo A . Environment impacts the metabolic dependencies of ras-driven non-small cell lung cancer. Cell Metab (2016) 23(3):517–28. doi: 10.1016/j.cmet.2016.01.007 PMC4785096 26853747

[53] Noy R Pollard JW . Tumor-associated macrophages: From mechanisms to therapy. Immunity (2014) 41(1):49–61. doi: 10.1016/j.immuni.2014.06.010 25035953 PMC4137410

[54] Qian B-Z Pollard JW . Macrophage diversity enhances tumor progression and metastasis. Cell (2010) 141(1):39–51. doi: 10.1016/j.cell.2010.03.014 20371344 PMC4994190

[55] Kim K Skora AD Li Z Liu Q Tam AJ Blosser RL . Eradication of metastatic mouse cancers resistant to immune checkpoint blockade by suppression of myeloid-derived cells. Proc Natl Acad Sci USA (2014) 111(32):11774–9. doi: 10.1073/pnas.1410626111 PMC4136565 25071169

[56] Lubas MJ Kumar SS . The combined use of sbrt and immunotherapy–a literature review. Curr Oncol Rep (2020) 22(12):117. doi: 10.1007/s11912-020-00986-9 32929678

[57] Aliru ML Schoenhals JE Venkatesulu BP Anderson CC Barsoumian HB Younes AI . Radiation therapy and immunotherapy: What is the optimal timing or sequencing? Immunotherapy (2018) 10(4):299–316. doi: 10.2217/imt-2017-0082 29421979 PMC5810851

[58] Shaverdian N Lisberg AE Bornazyan K Veruttipong D Goldman JW Formenti SC . Previous radiotherapy and the clinical activity and toxicity of pembrolizumab in the treatment of non-Small-Cell lung cancer: A secondary analysis of the keynote-001 phase 1 trial. Lancet Oncol (2017) 18(7):895–903. doi: 10.1016/S1470-2045(17)30380-7 28551359 PMC5538772

[59] Theelen WSME Peulen HMU Lalezari F van der Noort V de Vries JF Aerts JGJV . Effect of pembrolizumab after stereotactic body radiotherapy vs pembrolizumab alone on tumor response in patients with advanced non-small cell lung cancer: Results of the pembro-rt phase 2 randomized clinical trial. JAMA Oncol (2019) 5(9):1276–82. doi: 10.1001/jamaoncol.2019.1478 PMC6624814 31294749

[60] Bauml JM Mick R Ciunci C Aggarwal C Davis C Evans T . Pembrolizumab after completion of locally ablative therapy for oligometastatic non–small cell lung cancer a phase 2 trial. JAMA Oncol (2019) 5(9):1283–90. doi: 10.1001/jamaoncol.2019.1449 PMC6624820 31294762

[61] Seung SK Curti BD Crittenden M Walker E Coffey T Siebert JC . Phase 1 study of stereotactic body radiotherapy and Interleukin-2–tumor and immunological responses. Sci Transl Med (2012) 4(137):137ra74. doi: 10.1126/scitranslmed.3003649 22674552

[62] Yoneda K Kuwata T Kanayama M Mori M Kawanami T Yatera K . Alteration in tumoural pd-L1 expression and stromal Cd8-positive tumour-infiltrating lymphocytes after concurrent chemo-radiotherapy for non-small cell lung cancer. Br J Cancer (2019) 121(6):490–6. doi: 10.1038/s41416-019-0541-3 PMC6738061 31388183

[63] Djordjevic B Hennessy BT Li J Barkoh BA Luthra R Mills GB . Clinical assessment of pten loss in endometrial carcinoma: Immunohistochemistry out-performs gene sequencing. Mod Pathol (2012) 25(5):699–708. doi: 10.1038/modpathol.2011.208 22301702 PMC3341518

[64] Liu X Gong Y . Isocitrate dehydrogenase inhibitors in acute myeloid leukemia. biomark Res (2019) 7:22. doi: 10.1186/s40364-019-0173-z 31660152 PMC6806510

[65] Yuneva MO Fan TWM Allen TD Higashi RM Ferraris DV Tsukamoto T . The metabolic profile of tumors depends on both the responsible genetic lesion and tissue type. Cell Metab (2012) 15(2):157–70. doi: 10.1016/j.cmet.2011.12.015 PMC3282107 22326218

